# Functional consequences of A-to-I editing of miR-379 in prostate cancer cells

**DOI:** 10.1038/s41598-023-43775-7

**Published:** 2023-10-03

**Authors:** Gjendine Voss, James R. Cassidy, Yvonne Ceder

**Affiliations:** Division of Translational Cancer Research, Department of Laboratory Medicine, Lund University, Lund, Sweden

**Keywords:** Prostate cancer, RNA

## Abstract

Prostate cancer is the predominant cause of cancer in men, but there is still a lack of biomarkers and treatments for metastatic spread. The initial promise of microRNAs to provide avenues to solve these problems has been dampened by the realisation that microRNAs co-exist in multiple functionally distinct isoforms, for example due to A-to-I editing. We recently found that A-to-I-editing of microRNA-379 (miR-379) was associated with prostate cancer, and that only the unedited isoform was negatively correlated with aggressive disease. Here, we set out to decipher the biological effects of unedited and edited miR-379 in prostate cancer cells. After transfection of four different prostate cancer cell lines with isoform-specific miR-379 mimics, we performed assays for cell growth, colony formation, migration, cell–cell adhesion, and analysed epithelial–mesenchymal transition (EMT) and stemness markers. We found that unedited miR-379 affected cell growth, with a promoting function in androgen receptor (AR)-negative cells and an inhibiting effect in AR-positive cells. This is supported by our in silico analysis that found unedited miR-379 targets are predicted to be predominantly involved in cellular proliferation whereas the targets of edited miR-379 are not. We further found that both miR-379 isoforms could promote colony formation, migration, and cell–cell adhesion. Overall, our data suggests that editing of miR-379 attenuates the growth-suppressive function of unedited miR-379 in androgen-sensitive prostate cancer cells, thereby promoting tumor growth.

## Introduction

Prostate cancer accounts for approximately 25% of all new cancer diagnoses in European men^1^. When diagnosed in its early stages, the disease can be cured, or managed well for decades^2^. However, if the tumour progresses and metastasises, treatment options are limited, and patients eventually succumb to the disease. Hormone deprivation treatment can delay progression, but cannot cure the patient, and development of castration resistance is common^2^. A better understanding of the molecules involved in metastatic disease can also help to recognise the early steps of metastasis and enable intervention before metastatic spread occurs.

Several different pathways and molecules are known to be involved in the metastatic process. For example, epithelial–mesenchymal transition (EMT) leads to an increased migratory potential of cancer cells, which in turn promotes metastasis. At the metastatic site, the cells are thought to revert back through mesenchymal-epithelial transition (MET), which acts to support tumour formation^3^. However, there is also evidence that some cells do not fully complete the EMT program, but merely take on a hybrid state in which they express both epithelial and mesenchymal markers^4–6^.

These complex gene expression programs are not only controlled by regulatory proteins such as transcription factors, but also through non-coding RNAs. For example, the microRNA-200 family and microRNA-205 can skew cells towards an epithelial phenotype by suppressing ZEB1^7–9^. Similarly, microRNA-96 can promote epithelial features both by suppressing their translational repressor ZEB1^10,11^ and by directly upregulating E-Cadherin and EpCAM proteins^12^. The regulation by microRNAs (miRNAs) is mediated through imperfect sequence complementarity to the mRNA target. A single miRNA can have hundreds of different targets in a cell, some with overlapping or opposing functions, facilitating the fine-tuning of biological processes. The deregulation of miRNAs in diseases, including prostate cancer^9,13^, has been established for several years now and has inspired many efforts to implement these molecules as biomarkers or therapeutic targets^14,15^.

As RNA sequencing is becoming more and more affordable, and bioinformatic pipelines are improving rapidly, large-scale analysis of sequenced sample sets has revealed that RNA heterogeneity is much higher than previously anticipated^16–18^. For example, adenosine deaminase acting on RNA (ADAR) enzymes can convert adenosine nucleotides to inosines in the process of A-to-I editing^17–19^. All double-stranded RNAs in a cell represent potential ADAR targets, including primary miRNAs (pri-miRNAs) from which miRNAs are produced, which can affect both pri-miRNA processing and target selection of the resulting mature miRNA^20^. This forces us to re-evaluate the functions and clinical potential previously assigned to different miRNAs in an isoform-specific manner.

We recently developed an RT-qPCR method that can distinguish between A-to-I-edited miRNAs and determined the levels of each editing isoform of miR-379 in a clinical prostate cancer cohort^21,22^. The editing site of miR-379 is located in the seed sequence^23^, and the two isoforms have been proposed to bind different sets of mRNA targets^24^. Editing of pri-miR-379 has also been shown to impede miR-379 processing and therefore the levels of mature edited miR-379^23,25^. Editing of pri-miR-379 therefore does not substitute mature unedited miR-379 with the same number of mature edited miR-379 molecules, but will lead to an overall decrease in miR-379 levels.

We found that the miR-379 editing frequency was increased in patients with prostate cancer compared to those with benign prostatic hyperplasia. Interestingly, downregulation of unedited miR-379 was associated with metastasis, castration resistance and shorter overall survival, whereas edited miR-379 levels were not associated with these parameters^21^. It suggests that edited miR-379 may not have the same function as unedited miR-379 in prostate cancer cells.

The following three hypotheses were formed based on these results:

1. The main function of miR-379 editing is the downregulation of the miRNA. In this model, either only unedited miR-379 or both isoforms could have a tumour-suppressive function.
2. Increased editing of miR-379 leads to increased production of edited miR-379, which could have a tumour-promoting function.
3. Increased miR-379 editing and reduction of unedited miR-379 are merely the result of increased ADAR2 editing activity in prostate tumours, but play no active role in prostate cancer development.

It is also possible that both mechanisms stated in hypotheses 1 and 2 are at work, where increased miR-379 editing leads to both a reduction of putatively tumour-suppressive unedited miR-379, and an increase of potentially oncogenic edited miR-379. To collect more evidence to support or reject these hypotheses, we transfected different prostate cancer cell lines with unedited and edited miR-379 and performed different functional in vitro assays as well as gene expression analyses and in silico target predictions.

## Results

### The effect of miR-379 on cell growth differs in androgen receptor (AR)-positive and -negative cell lines

**Figure 1:**
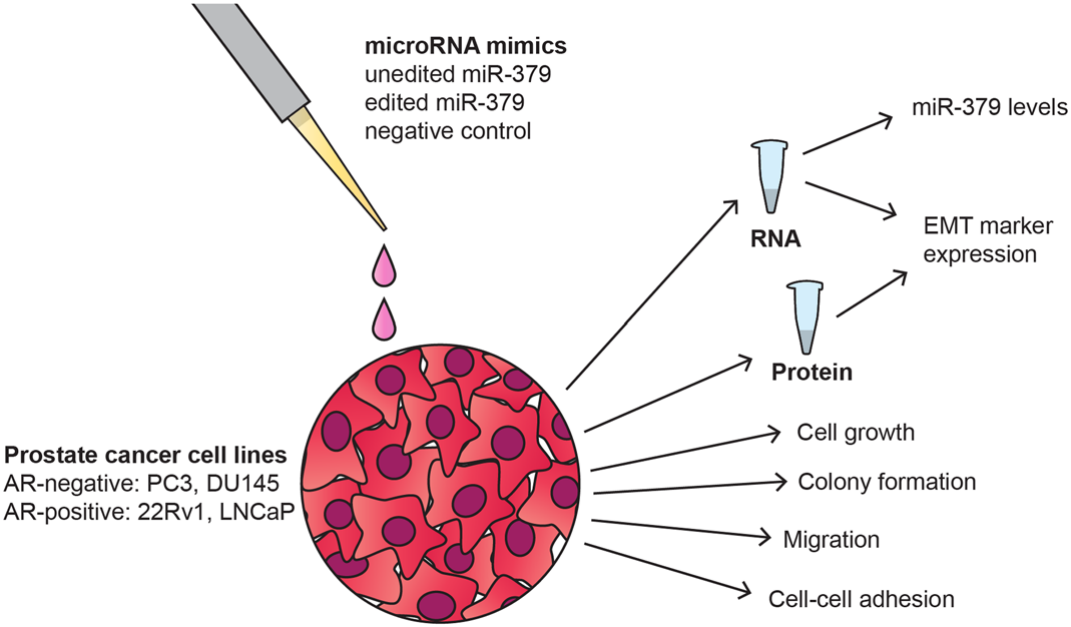
Study design. Four different prostate cancer cell lines were transiently transfected with mimics of unedited or edited miR-379 or negative control. Successful transfection was confirmed by isoform-specific miR-379 RT-qPCR. The cells were then assayed for cell growth, colony formation, and gene expression.

In order to characterise the role of editing isoforms of miR-379 in a range of prostate cancer cell lines, we selected two androgen receptor (AR)-negative cell lines (PC3 and DU145) and two AR-positive/androgen-responsive cell lines (22Rv1 and LNCaP)^26,27^. We transfected the cells with the two miR-379 isoforms or negative control mimics and performed a variety of in vitro assays to characterise biological functions that are relevant in the context of cancer (Fig. 1).

To confirm successful transfection of the cells, we performed isoform-specific RT-qPCR^21^ on RNA from transfected cells. In all cell lines, we found approximately 1000-fold upregulation of the transfected miR-379 isoform compared to negative control cells (Supplementary Fig. 1).

**Figure 2:**
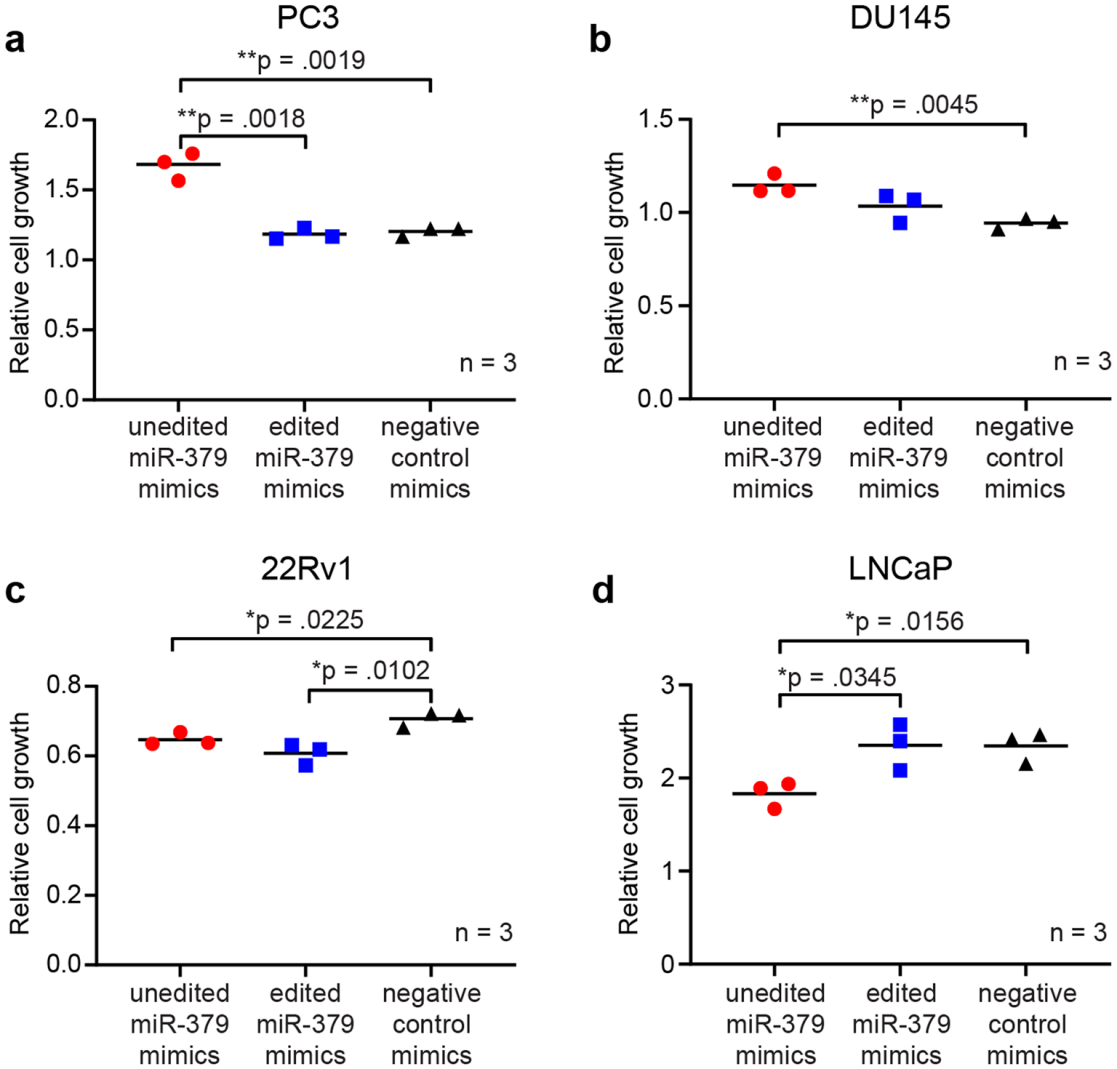
Cell growth in miR-379-transfected cells. SRB assays to measure relative cell growth were performed 96 h after transfection with unedited miR-379 (red circles), edited miR-379 (blue squares) or negative control mimics (black triangles). Results are shown for androgen-independent cell lines PC3 (**a**) and DU145 (**b**), and androgen-sensitive cell lines 22Rv1 (**c**) and LNCaP (**d**). The experiment was repeated at least three times in each cell line, and representative figures are presented. Individual values of biological triplicates are shown; the line denotes the mean. The absorbance after staining is indicative of cell mass. Unpaired two-tailed Student’s *t*-tests were performed to compare the treatment groups to one another; *p < 0.05, **p < 0.01. Only statistically significant *p* values are shown in the figure.

When analysing cell growth using Sulforhodamine-B (SRB) assays, we observed different effects of miR-379 isoforms depending on the AR status of the cell line. PC3 and DU145 cells, which do not express AR, both showed increased cell growth (p = 0.0019 and p = 0.0045 respectively) upon transfection with unedited miR-379 (Fig. 2a,b), whereas edited miR-379 did not give a statistically significant effect on the cells. On the contrary, in the AR-positive androgen-responsive cell lines 22Rv1 and LNCaP^26^ (Fig. 2c,d), unedited miR-379 inhibited cell growth (p = 0.0225 and p = 0.0156 respectively). In 22Rv1 cells, edited miR-379 had the same effect as unedited miR-379 (p = 0.0102), whereas in LNCaP cells, cells treated with edited miR-379 did not grow more or less than control cells.

Based on these results, unedited miR-379 seems to be the only isoform with a strong effect on cell growth, and this effect is opposite in our androgen-responsive and unresponsive model systems.

### Edited miR-379 enhances colony formation and migration

**Figure 3:**
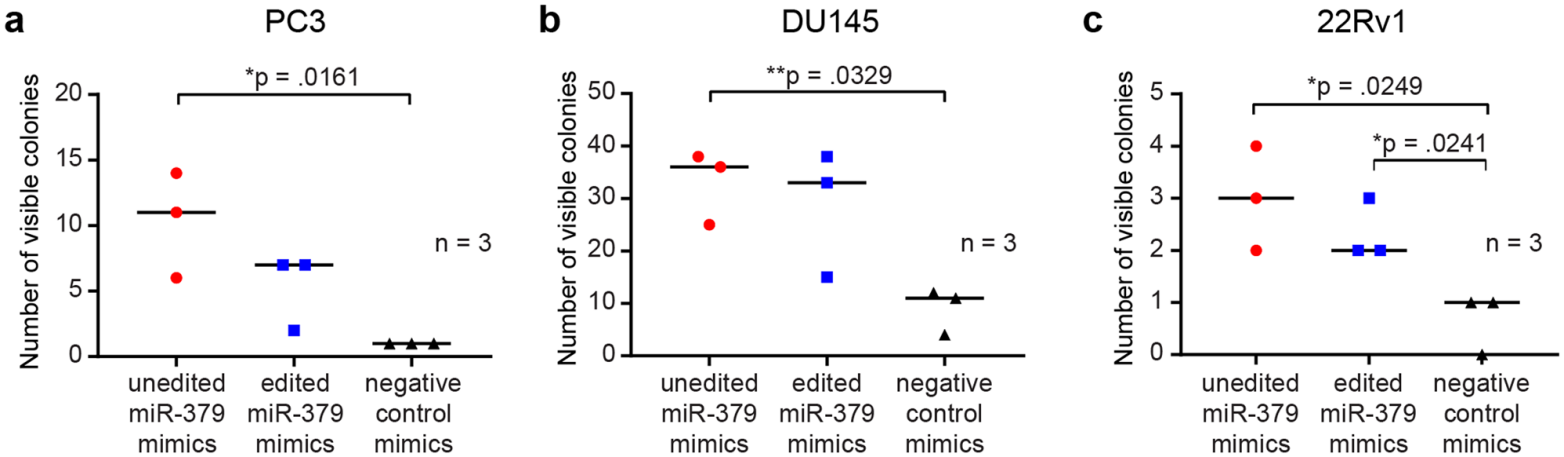
Colony formation potential in miR-379-transfected cells. Anchorage-independent colony formation assays were performed following transfection with unedited miR-379 (red circles), edited miR-379 (blue squares) or negative control mimics (black triangles) and visible colonies were counted. Results are shown for PC3 (**a**), DU145 (**b**) and 22Rv1 (**c**) cells. Experiments were repeated three times, and representative data are shown. Individual values of biological triplicates are shown; the line denotes the mean. Unpaired two-tailed Student’s *t*-tests were performed to compare the treatment groups to one another; *p < 0.05, **p < 0.01. Only statistically significant *p* values are shown in the figure.

Next, we assessed the colony formation capacity of miR-379-transfected cells using anchorage-independent soft agar assays. Unedited miR-379 enhanced colony formation in PC3, DU145 and 22Rv1 (p = 0.0161, p = 0.0329 and p = 0.0249 respectively), and the same trend was seen for edited miR-379 (Fig. 3) but it was only significant in the 22Rv1 cell line (p = 0.0241). LNCaP cells were unable to form colonies independently of the transfected mimic.

**Figure 4:**
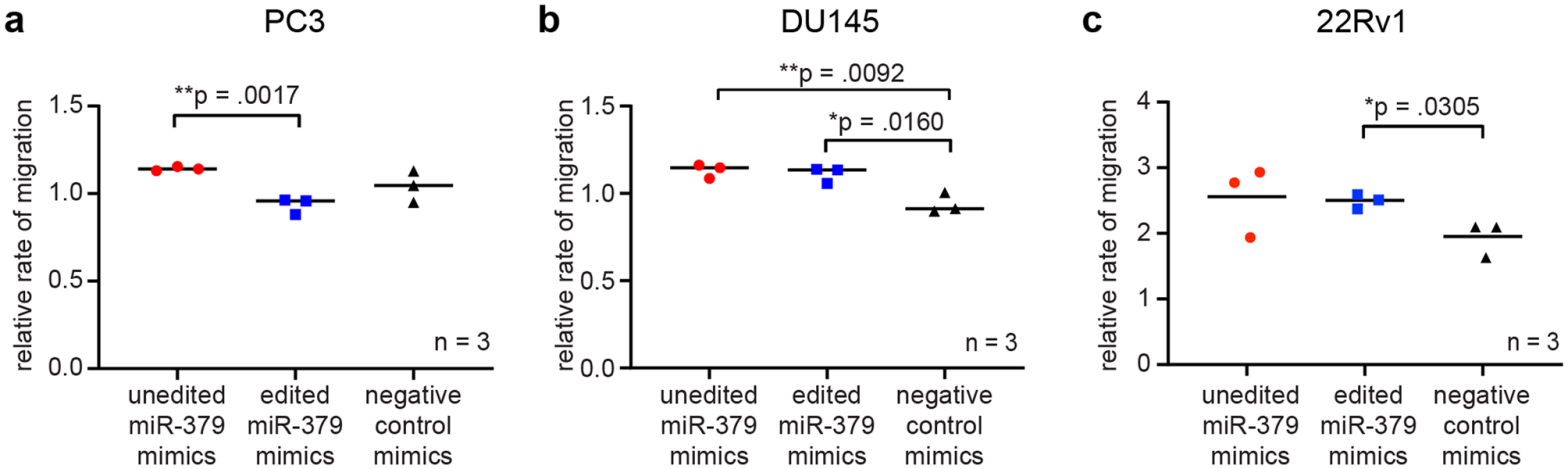
Migration potential in miR-379-transfected cells. Migration was assessed using Boyden chambers 24 h after transfection with unedited miR-379 (red circles), edited miR-379 (blue squares) or negative control mimics (black triangles). Results are shown for PC3 (**a**), DU145 (**b**) and 22Rv1 (**c**) cells. Experiments were repeated three times, and representative data are shown. Individual values of biological triplicates are shown; the line denotes the mean. Unpaired two-tailed Student’s *t*-tests were performed to compare the treatment groups to one another; *p < 0.05, **p < 0.01. Only statistically significant *p* values are shown in the figure.

We also performed Boyden chamber migration assays. In DU145 cells, a statistically significant increase in migration ability upon transfection with unedited miR-379 was observed (p = 0.0092), a similar but non-significant trend could be seen in PC3 and 22Rv1 cells as well (Fig. 4). An increase in cell migration was also observed in DU145 and 22Rv1 cells transfected with edited miR-379 (p = 0.0160 and p = 0.0305 respectively).

### Gene expression analysis of EMT and stemness markers

The observed effects of miR-379 on colony formation prompted us to assess stemness markers. To this end, we performed qPCR on *CD44*, *KIT* and *NANOG* mRNAs (Supplementary Fig. 2). The expression of *CD44* mRNA was slightly lower in cells transfected with miR-379 mimics. Although there were some differences for individual mRNAs, implying some biological effect, there was no overall pattern among these stemness markers.

Next, we asked whether some of the phenotypic changes observed could be the result of EMT. We therefore investigated gene expression of a selection of mRNAs whose gene products are commonly associated with EMT (Supplementary Fig. 3). Overall, there was little evidence on the transcriptional level that would suggest that a large-scale EMT or MET program is induced by transfection with either unedited or edited miR-379.

**Figure 5:**
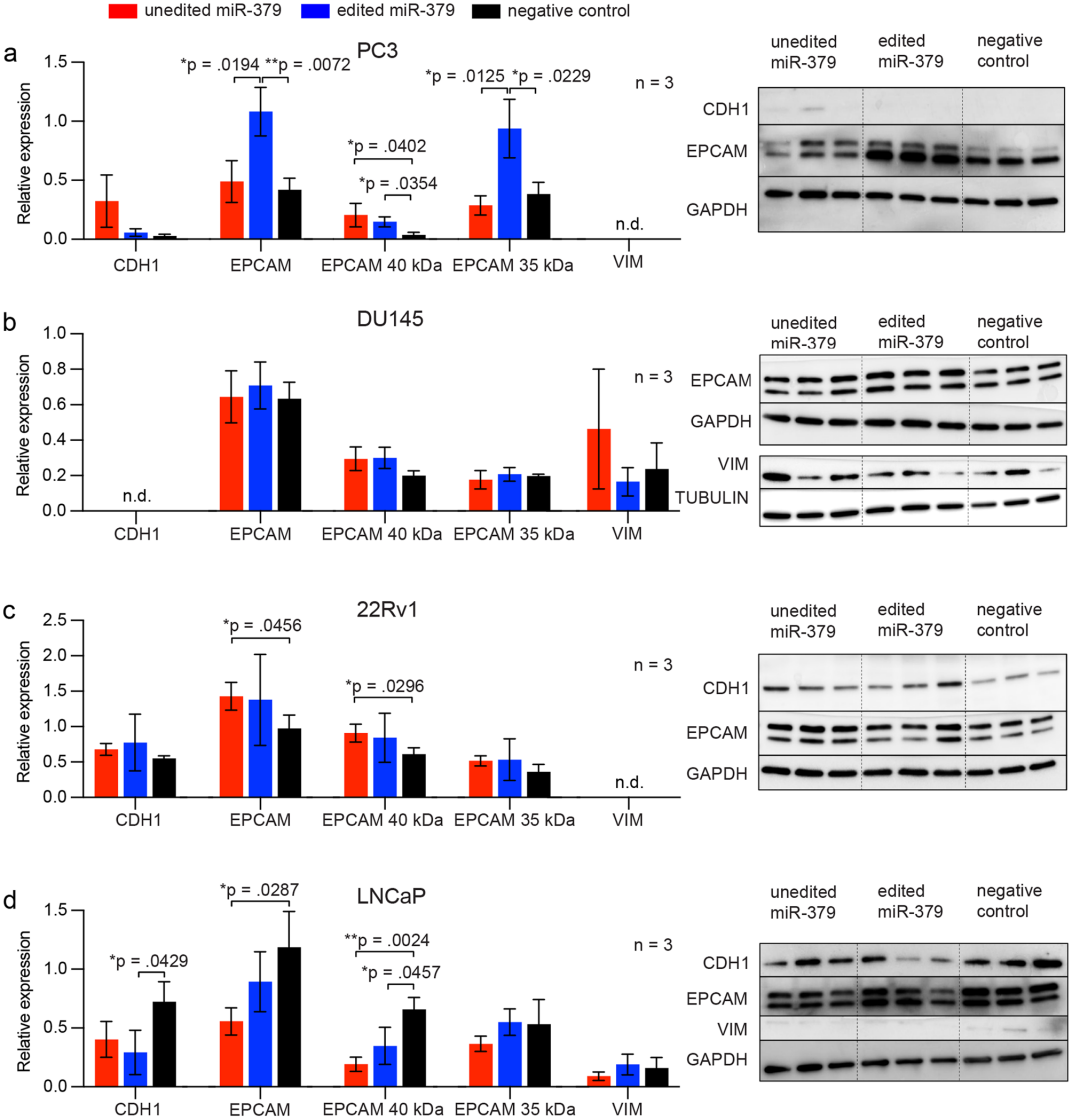
Protein expression of epithelial and mesenchymal markers. PC3 (**a**), DU145 (**b**), 22Rv1 (**c**) and LNCaP (**d**) cells were transfected with miR-379 mimics for 48 h before protein isolation and Western blotting. Expression of CDH1, EPCAM (total, 40 kDa isoform and 35 kDa isoform) as well as Vimentin was normalised to the expression of GAPDH using densitometry. For Vimentin expression in DU145 (**b**) normalisation was done to Tubulin. Protein bands used for analysis are shown from PC3 (**a**), DU145 (**b**), 22Rv1 (**c**) and LNCaP (**d**) lysates. Experiments were repeated three times, and representative data are shown. Mean ± SD of triplicates is shown. Unpaired Student’s *t*-tests were performed to compare the treatment groups to one another; *p < 0.05, **p < 0.01. Only statistically significant *p* values are shown in the figure.

As the mRNA level does not necessarily correspond to the level of translated protein, we next moved to confirm these results on the protein level. There was no clear change upon miR-379 transfection for CDH1 nor vimentin expression in any of the cell lines (Fig. 5). In PC3 cells, EpCAM expression was higher in the cells transfected with edited miR-379 (p = 0.0072), consistent with what was seen at the transcriptional level. Interestingly, there seemed to be an isoform shift in relative EpCAM protein expression depending on editing status of miR-379 in PC3 cells. Transfection with edited miR-379 resulted in a significantly higher level of the 35 kDa isoform of EpCAM compared to cells transfected with unedited miR-379 (p = 0.0125) and negative control cells (p = 0.0229), but not the 40 kDa isoform, suggesting that editing of miR-379 may affect protein glycosylation. Levels of the 40 kDa isoform were statistically significantly higher in miR-379-transfected cells compared to the negative control, independently of editing status (p = 0.0402 for unedited and p = 0.0354 for edited).

Deglycosylation of lysates resulted in a singular protein band, confirming that the two isoforms were indeed a result of differing states of glycosylation (Supplementary Fig. 4).

### miR-379 increases cell–cell adhesion

**Figure 6:**
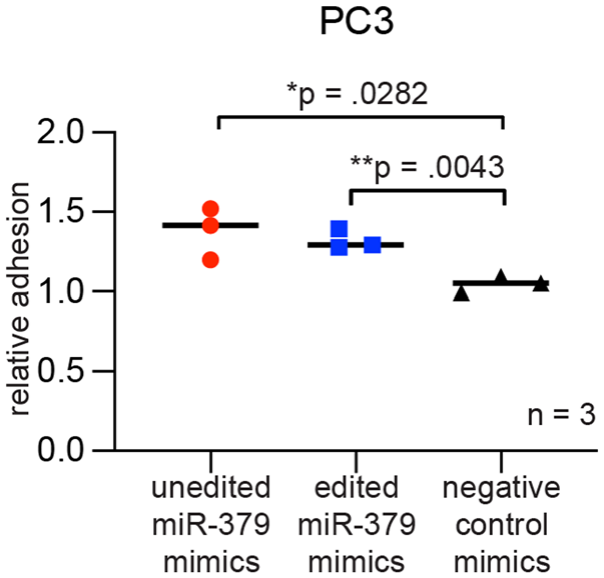
Cell–cell adhesion in miR-379-transfected PC3 cells. Adhesion assays were performed 3 days after transfection with unedited miR-379 (red circles), edited miR-379 (blue squares) or negative control mimics (black triangles). Experiments were repeated three times, and representative data is shown. Individual values of biological triplicates are shown; the line denotes the mean. Unpaired two-tailed Student’s *t*-tests were performed to compare the treatment groups to one another; *p < 0.05, **p < 0.01. Only statistically significant *p* values are shown in the figure.

The observed isoform shift of EPCAM in the PC3 cell line led us to test whether this change had a functional impact on cell adhesion. We performed cell–cell adhesion experiments and found there to be statistically significantly higher adhesion between PC3 cells transfected with both edited and unedited miR-379 mimics than the negative control (p = 0.0282 for unedited and p = 0.0043 for edited). This was consistent with the changes in protein levels of the more stable, fully glycosylated 40 kDa isoform of EpCAM^28^. (Fig. 6).

### In silico target prediction

**Figure 7:**
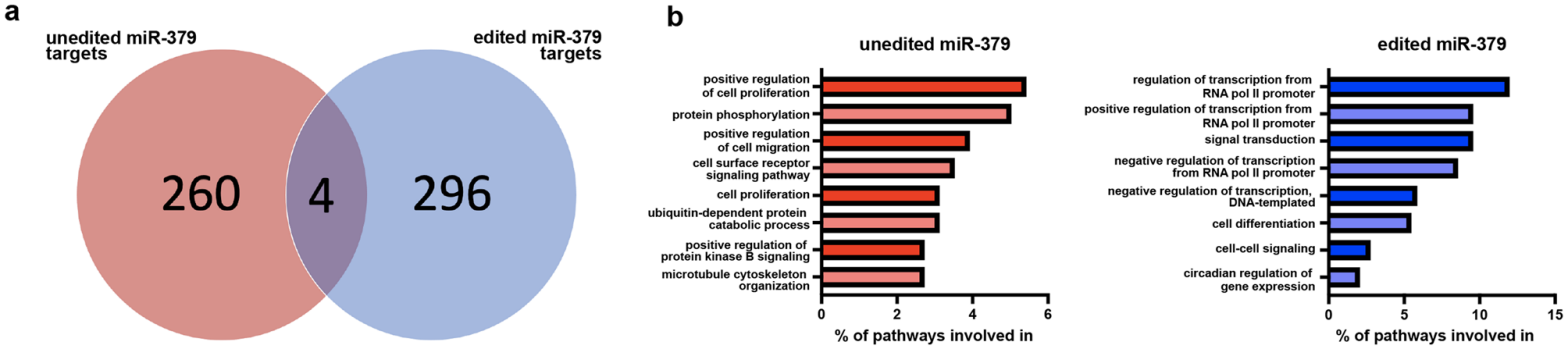
Predicted targets of unedited and edited miR-379. Targets of unedited and edited miR-379 were predicted using miRDB.org. The number of overall targets specifically for unedited (red) and edited (blue) are shown as well as 4 targets in common (**a**). The percentage of pathways that targets were involved in was determined by the online DAVID Bioinformatics website. Top 8 pathways are displayed for both unedited (red bars) and edited (blue bars) miR-379 (**b**).

As some of the biological effects of unedited and edited miR-379 differed, we performed an in silico target prediction and found 264 targets for unedited and 300 for edited with only four overlapping targets (Fig. 7a). The overlapping genes were pyruvate dehydrogenase kinase 1 (PDK1), protein tyrosine kinase 2 (PTK2), AP2 associated kinase 1 (AAK1) and nuclear factor of activated T cells 3 (NFATC3). A gene ontology analysis of the predicted targets found that unedited miR-379 targets were mostly enriched in proliferation, in line with the findings that only unedited miR-379 showed an effect on cell growth. The predicted targets for edited miR-379 targets were enriched in transcription regulation and signal transduction (Fig. 7b).

## Discussion

The goal of this study was to determine the functional consequences of editing of miR-379 in prostate cancer cells, characterise phenotypic changes caused by the different miR-379 isoforms, and to potentially identify large-scale pathways that may lead to these phenotypic changes.

First, we investigated the effect of unedited and edited miR-379 mimics on prostate cancer cell growth. Overall, only unedited miR-379 gave a clear phenotype, whereas edited miR-379 either had the same or a weaker effect as unedited miR-379, or no effect at all. Intriguingly, the effect of unedited miR-379 was dependent on the AR status of the cells, with a growth-promoting role in AR-negative and a growth-suppressive role in the investigated androgen-responsive cell lines. This suggests that the putatively tumour-suppressive function of unedited miR-379 in prostate cancer cells may be mediated through or in conjunction with the androgen signalling pathway. In line with this Gururajan et al*.* showed that decreased levels of miR-379 inhibited cell growth in the cell line ARCaP, the growth of which is inhibited by AR signaling^29,30^. This is especially interesting in the context of the only other study that has investigated the biological effect of miR-379 editing in cancer: based on various cancer cell lines, not including prostate cancer cells, the authors concluded that unedited, but not edited, miR-379 had a tumour-promoting effect and enhanced cell growth^24^. This is what we observed in PC3 and DU145 cells—but the opposite of what we found in the androgen-responsive cell lines. If unedited miR-379 has a tumour-suppressive role only in cells that respond to androgen signalling, this would explain why this effect was not observed in other androgen-independent cancer types. This idea is supported by a large-scale bioinformatic analysis of the TCGA dataset^31^. There, miR-379 editing was reduced in most cancer types compared to normal tissues, but increased in prostate cancer, which is in line with our findings^21^. While our results hint at a link between unedited miR-379 and androgen signalling, the underlying pathways and molecules will need to be studied in much greater detail in future studies. Androgens are the main growth factor for prostate cells, and while we found the effect of miR-379 on proliferation to depend on AR status, this difference was not seen for the other biological functions that we tested, which are less dependent on androgen signalling.

Aside from the apparent AR-dependence, the effect of miR-379 on cell growth also seemed to depend on editing status: for most of the tested cell lines, transfection with edited miR-379 did not change cell growth compared to the negative control. This difference in mechanism could be due to the different sets of targets for unedited and edited miR-379. The predicted targets of unedited miR-379 were enriched in proliferation, while the predicted targets of edited miR-379 had more general functions such as transcription regulation. Due to the large number of potential miRNA targets in different settings, the analysis of individual targets can be misleading. Each miRNA is expected to fine-tune hundreds of mRNAs at different affinities^32^, where the effects on each individual target may be small, but collectively skew the biology of the entire cell towards a phenotypic shift. Our results from the analyses of EPCAM glycosylation isoforms also suggest that editing of miRNAs can affect posttranslational modifications of proteins.

A limitation of the present study is that it is performed in vitro. However, stable overexpression of the miRNA would be required for in vivo long-term experiments. To this end, shRNA-like expression vectors encoding the miRNA precursor are commonly used^33^, but there is no evidence to support that this precursor would be edited, so that stable expression of edited miR-379 is not currently possible. Other groups have chosen to simply encode guanosine instead of the adenosine^24^. However, as target selection of miRNAs is affected by subtle differences in binding affinities, and the base pairing properties of guanosine and inosine are not identical^34^, A-to-G substitution is not a good model for studying the effects of edited miR-379.

Initially, we suggested three hypotheses for the biological effects of miR-379 editing. The findings described here support hypothesis 1, in which the main effect of increased miR-379 editing lies in reducing unedited miR-379 or miR-379 levels overall, and edited miR-379 either has no function or the same function as unedited miR-379. In general, our results indicate that edited miR-379 is a less potent isoform of the microRNA when it comes to cell growth but has the same general effect on other biological functions such as migration and cell–cell adhesion. This leads us to reject hypothesis 2 that proposed a distinct role for edited miR-379. Furthermore, the results presented here allow us to reject the null hypothesis that miR-379 plays no role relevant for prostate cancer development (hypothesis 3). This does however not prove that miR-379 editing or deregulation is necessarily a driver event; it may be one of many events contributing to an overall shift in the biology of a prostate cancer cell. As miR-379 is edited by ADAR2^21,23^, and ADAR2 also has multiple other substrates, miR-379 editing is likely not an isolated event, but one of many affected pathways.

Collectively, we postulate that the primary functional consequence of increased miR-379 editing in prostate cancer^21,31^ is the reduced expression of the unedited miR-379 isoform, which has a more potent role in suppressing cell growth in androgen-responsive cells. In our previous study using prostate cancer patient samples, we did indeed find a negative association between miR-379 editing levels and total miR-379 levels^21^ likely owing to the less efficient processing and maturation of edited pri-miR-379^23^. Understanding whether miR-379 editing or deregulation is essential for prostate cancer progression and the relation to androgen signalling should be the subject of future studies, with the ultimate end goal to determine the therapeutic potential of miR-379 mimics.

## Materials and methods

### Cell lines and transfection with miRNA mimics

PC3, DU145, 22Rv1 and LNCaP cells were purchased from the American Tissue Culture Collection and regularly authenticated, most recently in February 2023 by Eurofins Genomics. Cell lines were cultured according to the manufacturer’s specifications in media containing 10% FBS and 1% Penicillin–Streptomycin.

Double-stranded miRNA mimics for unedited miR-379, edited miR-379 and negative control (based on cel-miR-67) were designed as custom miRNA mimics with ON TARGET modifications on the passenger strand (Horizon Discovery, Cambridge, United Kingdom). The sequences of the mimics are shown in Supplementary Table 1. Cells were transfected with the mimics at a concentration of 120 nM using Oligofectamine (Invitrogen, Carlsbad, CA) according to the manufacturer’s instructions.

### RNA extraction

Total RNA was extracted from the cells using TRIzol reagent (Ambion, Carlsbad, CA) according to the manufacturer’s instructions either 24 h after transfection for measurement of miR-379 or 48 h after transfection for measurement of mRNA expression levels. RNA concentrations were determined using a NanoDrop 2000 spectrophotometer (Thermo Scientific, Wilmington, DE).

### RT-qPCR for miRNA expression

Quantification of miR-379 isoforms was done according to Voss et al*.*, using the same primer sequences^21,22^. Briefly, 1 µg total RNA was reverse transcribed using the qScript Flex kit (#95049-100, Quantabio, Beverly, MA) with 2 µl 5 × reaction mix, 1 µl GSP enhancer, 0.05 µM two-tailed RT primer, and 0.5 µl reverse transcriptase in a total volume of 10 µl. Reverse transcription was performed at 25 °C for 1 h, stopped at 85 °C for 5 min and samples held at 4 °C. qPCR was performed using PowerUp SYBR Green Master Mix (#A25742, Thermo Scientific, Vilnius, Lithuania) using 400 nM forward and reverse primers. The RT product constituted up to one tenth of the total qPCR reaction volume. Samples were assayed in triplicates using the QuantStudio 7 Flex qPCR machine (Applied Biosystems) with a qPCR program consisting of 30 s initial denaturation at 95 °C, followed by 45 cycles of 5 s at 95 °C and 20 s at 60 °C. TaqMan microRNA assays (Applied Biosystems, Pleasanton, CA) were used for small RNA housekeeping controls according to the manufacturer’s instructions with 100 ng RNA input. The geometric mean of RNU44 (#001094) and RNU48 (#001006) was used for normalization of miR-379 expression using the ΔCt method.

### Gene expression analysis

Total RNA was treated with DNase I (Thermo Scientific, Vilnius, Lithuania) according to the manufacturer’s instructions at 37 °C for 30 min. Total cDNA from 3 µg RNA was synthesised using the RevertAid H Minus First Strand cDNA Synthesis kit (#K1632, Thermo Scientific) according to the manufacturer’s instructions. The cDNA was diluted 1:5 and 1 µl of the diluted cDNA was used for qPCR in triplicates. TaqMan Gene expression assays and TaqMan Gene expression Master Mix (#4369016, Thermo Scientific) were used according to the manufacturer’s instructions on a QuantStudio 7 Flex machine. Expression of *CDH1* (Hs01023895_m1), *EPCAM* (Hs00158980_m1), *OCLN* (Hs05465837_g1), *VIM* (Hs00958111_m1), *SNAI1* (Hs00195591_m1), *ZEB1* (Hs01566408_m1), *CD44* (Hs01075861_m1), *KIT* (Hs00174029_m1) and *NANOG* (Hs04399610_g1) mRNAs was normalised to the geometric mean of *GUSB* (Hs00939627_m1), *PGK1* (Hs00943178_g1) and *GAPDH* (Hs02758991_g1) mRNAs using the ΔCt method.

### SRB assay for cell growth

For cell growth assays, cells were seeded in 6-well plates and transfected with miR-379 mimics as described above. After 96 h, the cells were fixed with ice-cold 10% TCA and washed with tap water. Cells were stained with 500 µl 0.4% SRB (Sigma-Aldrich, St. Louis, MO) in 1% acetic acid for 15 min at room temperature, and unbound SRB was washed away with 1% acetic acid. After drying the plates, bound SRB was dissolved in 500 µl Tris base. Triplicates of 100 µl were transferred onto a microplate for absorbance measurement using a Synergy 2 plate reader (BioTek, Winooski, VT). The absorbance at 690 nm was subtracted from absorbance values at 490 nm to determine relative cell mass in each well.

### Colony formation assay

Colony formation assays were performed in 6-well plates. Briefly, the base layer was prepared as 2 ml complete medium with 0.5% standard agarose (Saveen & Werner, Malmö, Sweden) and left to set at room temperature. Cells that had been transfected with miR-379 mimics the previous day were prepared in 2 ml appropriate media containing 0.3% standard agarose and were seeded to give 1000 cells per well. After the agarose set, medium was added to prevent drying of the agarose and replenished regularly over the course of the experiment. When colonies were visibly present, they were fixed in ice-cold methanol for 10 min and stained with 0.01% crystal violet (Sigma-Aldrich) in 20% methanol for 1 h. Colonies were washed with tap water until the background was clear and the number of colonies was manually counted.

### Migration assay

Cell migration was assessed using the Boyden chamber method in a 6-well plate format. One day after transfection with mimics, cells were made up to a concentration of 1 × 10^5^ cells/ml in serum-free media. From this solution, 1 ml was added inside of the transwell insert (Corning, Kennebunk, ME) and 2 ml of appropriate media with 10% FBS was added to the well outside of the insert. The cells were fixed with methanol after 20 h and stained with 0.1% Crystal Violet (Sigma-Aldrich) solution in 20% methanol for 15 min. Unbound Crystal Violet was removed by washing with tap water and cells that had not migrated were removed by swabbing the inside of the inserts with cotton buds. The migrated cells were dissolved in 1.5 ml 10% acetic acid. Triplicates of 100 µl were transferred onto a microplate and absorbance read at 595 nm using a Synergy 2 plate reader (BioTek, Winooski, VT). The results were normalised to control wells containing serum-free media on the outside of the insert.

### Adhesion assay

Adhesion assays were performed in a 24-well format 3 days after transfection with miR-379 mimics. Monolayer of wild type cells were seeded in the 24-well plate the day before the experiment by seeding 100,000 cells to each corresponding well. In brief transfected cells were detached with Versene (Thermo Scientific) and resuspended in PBS before being spun down at 300*×g* for 5 min and then resuspended in 1 ml of BCECF (#B3051, Fisher Scientific, Eugene, OR) and incubated at 37 °C for 15 min. Cells were spun down at 300*×g* for 5 min again and resuspended in appropriate medium to get a final concentration of 8 × 10^4^ cells/ml. 500 µl of this cell suspension was added to the monolayer of cells and incubated at 37 °C for 3 h. Afterwards media was removed and 100 µl 5 × passive lysis buffer (Promega, Madison, WI) was added. The plate was incubated at room temperature for 15 min on an orbital shaker before 45 µl duplicates were transferred to a 96-well plate and fluorescence was measured (excitation 490 nm, emission 535 nm). The results were normalised to an aliquot of the suspension of stained cells that was added to the 24-well plate originally, treated with passive lysis buffer.

### Western blots

Lysates of the miR-379-transfected cells were gathered using M-PER lysis buffer (Thermo Scientific) 2 days after transfection, and protein concentrations were determined using Coomassie Bradford assay (Thermo Scientific, Rockford, IL). In total 20 µg protein was combined with 4 × Laemmli buffer (BioRad) and incubated for 95 °C for 5 min. After this the lysate solution was loaded into a 4–20% Mini-PROTEAN^®^ TGX™ gel (BioRad) along with a protein ladder and run until bands had reached the bottom of the gel. The gels were transferred to a membrane using the Trans-Blot Turbo system (BioRad) and the membrane was then blocked at 4 °C with a 5% milk in PBST. The membrane was incubated overnight at 4 °C with the appropriate primary antibody in 5% milk in PBST. The primary antibodies used were; anti-CDH1 (sc-8426, Santa Cruz Biotechnology, Dallas, TX; 1:500), anti-Vimentin (ab20346, abcam, Cambridge, UK;1:1000), anti-EPCAM (sc-25308, Santa Cruz Biotechnology; 1:500), anti-GAPDH (MAB374, Merck Millipore, Darmstadt, Germany; 1:10,000). To save time and unnecessary stripping of the membranes which leads to increased protein degradation the membranes were cut before primary antibodies were added. Membranes were imaged using the Amersham imaging machine Amersham Imager 600 (GE Healthcare), and ImageJ was used for densitometry. Deglycosylation of lysates was done using PNGase F from *Elizabethkingia meningoseptica* (Sigma-Aldrich). In brief, 2 µl of the PNGase F solution was added to 20 µl of the protein lysates followed by incubation at 37 °C for 18 h. The treated lysates were run on the gel as described above.

### Data processing and statistical analysis

miRNA target predictions were performed in miRDB^35^ (http://www.mirdb.org/custom.html, accessed the 16th of august 2023). The A-to-I edited nucleotide in the edited version of miR-379 was entered as a G. Gene ontology analysis was performed using DAVID^36^ (http://david.abcc.ncifcrf.gov, accessed the 16th of august 2023). GraphPad Prism 9 (GraphPad software, La Jolla, CA) was used to perform all statistical analyses. All experiments were performed in triplicates, and groups were compared using two-tailed unpaired Student’s *t*-tests, with a significance level of α = 0.05.

### Supplementary Information

Supplementary Information.

## Data Availability

All data generated or analysed during this study are included in this published article, and its supplementary information files.
