# Supplementary material for: Functional consequences of A-to-I editing of miR-379 in prostate cancer cells
Source: Sci Rep. 2023 Oct 3;13:16602. doi: 10.1038/s41598-023-43775-7 (PMC10547749; doi:10.1038/s41598-023-43775-7)
Supplement: Supplementary file 1 — Supplementary Information. [file 41598_2023_43775_MOESM1_ESM.docx]

**
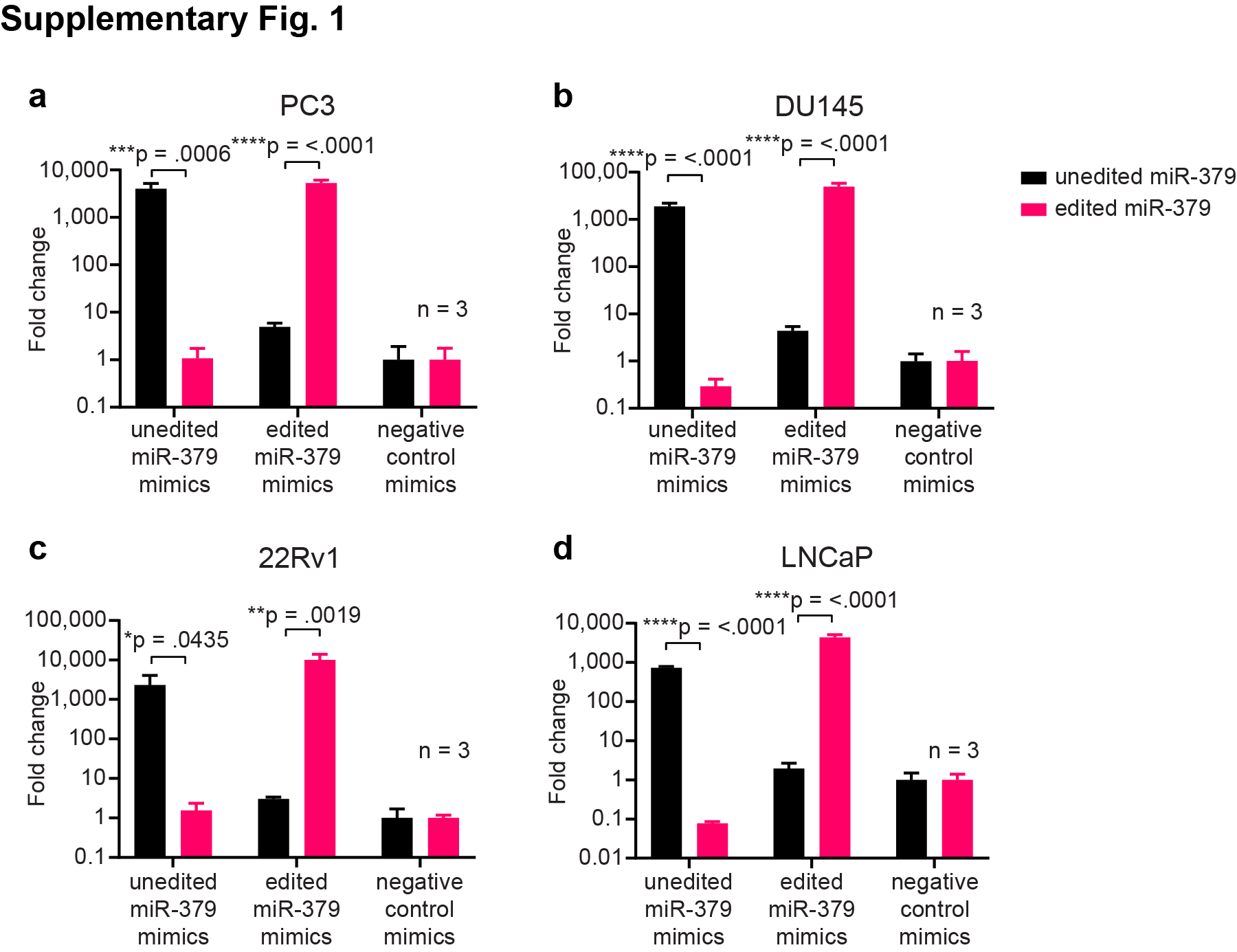
Supplementary Figure 1.** miR-379 levels in transfected cells. PC3 (**a**), DU145 (**b**), 22Rv1 (**c**) and LNCaP (**d**) prostate cancer cells were transfected with unedited miR-379, edited miR-379 or negative control mimics. RNA was isolated after 24 h and levels of unedited miR-379 (black bars) and edited miR-379 (pink bars) were measured using isoform-specific RT-qPCR. Expression of miR-379 was normalised to the geometric mean of RNU44 and RNU48, and the fold change was calculated relative to cells transfected with the negative control. Mean + SD of triplicates are shown. Unpaired two-tailed Student’s *t*-tests were performed, and asterisks above bars indicate that values in this treatment group reached the denoted threshold of statistical significance in direct comparisons with both other treatment groups; **p* < 0.05, ***p* < 0.01; ****p* < 0.001; *****p* < 0.0001. Only statistically significant *p* values are shown in the figure.

**
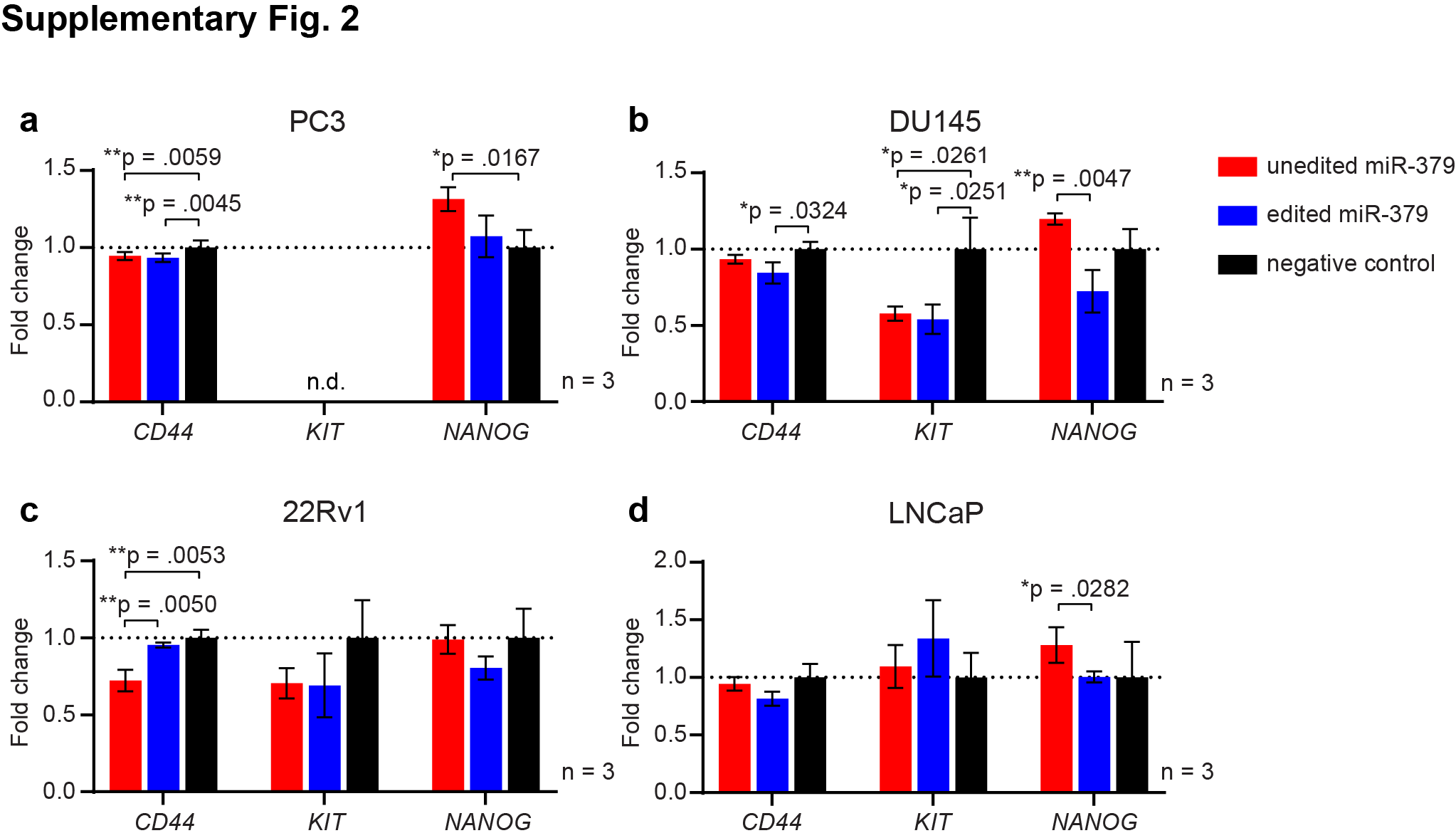
Supplementary Figure 2.** Gene expression of stemness markers. PC3 (**a**), DU145 (**b**), 22Rv1 (**c**) and LNCaP (**d**) cells were transfected with miR-379 mimics for 48 h before RNA isolation and qPCR using TaqMan assays. Expression of *CD44*, *KIT* and *NANOG* mRNAs was normalised to the geometric mean of *GUSB*, *PGK1* and *GAPDH* mRNAs. Experiments were repeated three times and representative data are shown. Mean ± SD of triplicates is shown. Unpaired two-tailed Student’s *t*-tests were performed to compare the treatment groups to one another; **p* < 0.05, ***p* < 0.01; ****p* < 0.001; *****p* < 0.0001. Only statistically significant *p* values are shown in the figure.


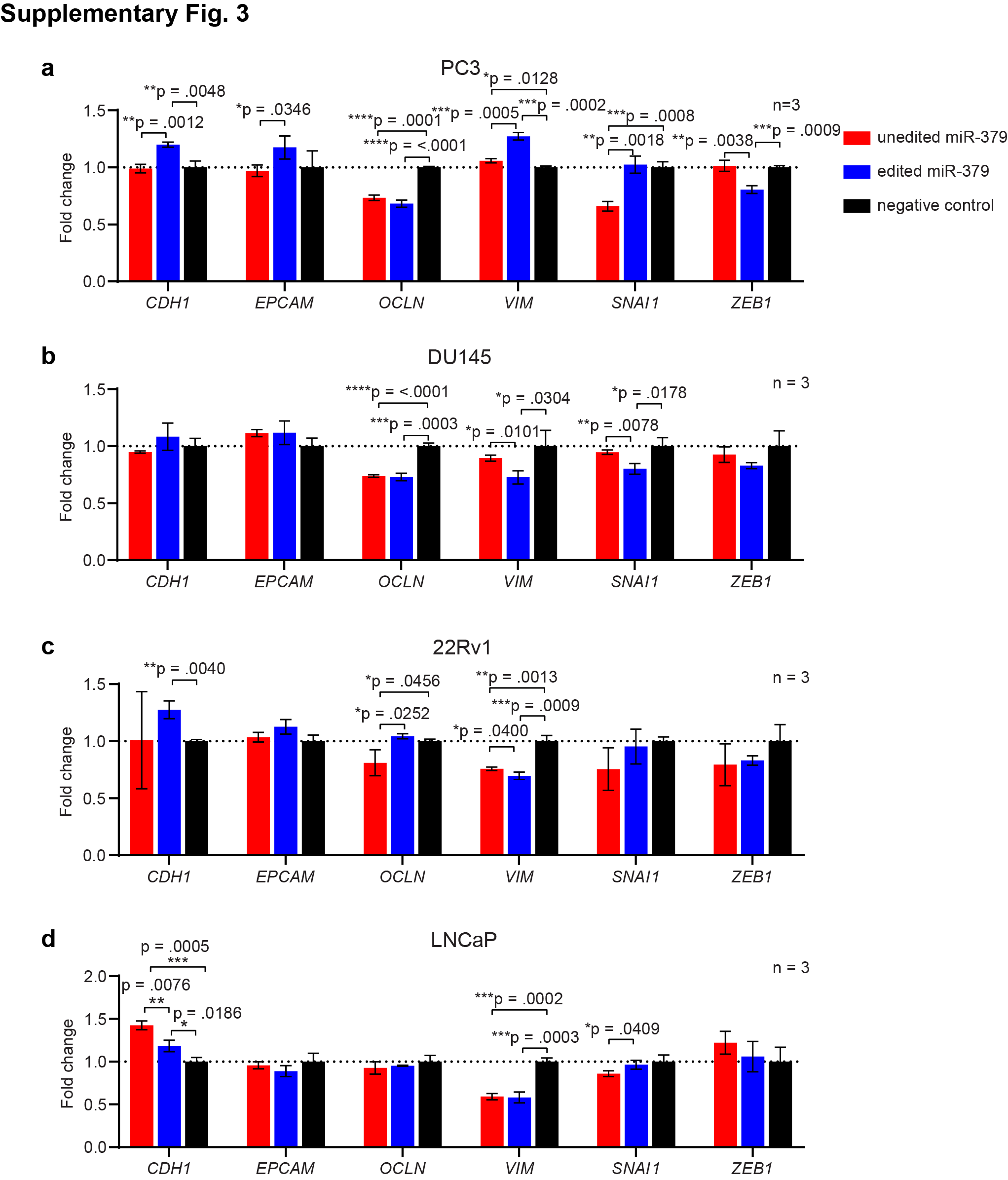


**Supplementary Figure 3.** Gene expression of epithelial and mesenchymal markers. PC3 (**a**), DU145 (**b**), 22Rv1 (**c**) and LNCaP (**d**) cells were transfected with miR-379 mimics for 48 h before RNA isolation and qPCR using TaqMan assays. Expression of *CDH1*, *EPCAM*, *OCLN*, *VIM*, *SNAI1* and *ZEB1* mRNAs was normalised to the geometric mean of *GUSB*, *PGK1* and *GAPDH* mRNAs. Experiments were repeated three times and representative data are shown. Mean ± SD of triplicates is shown. Unpaired two-tailed Student’s *t*-tests were performed to compare the treatment groups to one another; **p* < 0.05, ***p* < 0.01; ****p* < 0.001; *****p* < 0.0001. Only statistically significant *p* values are shown in the figure.


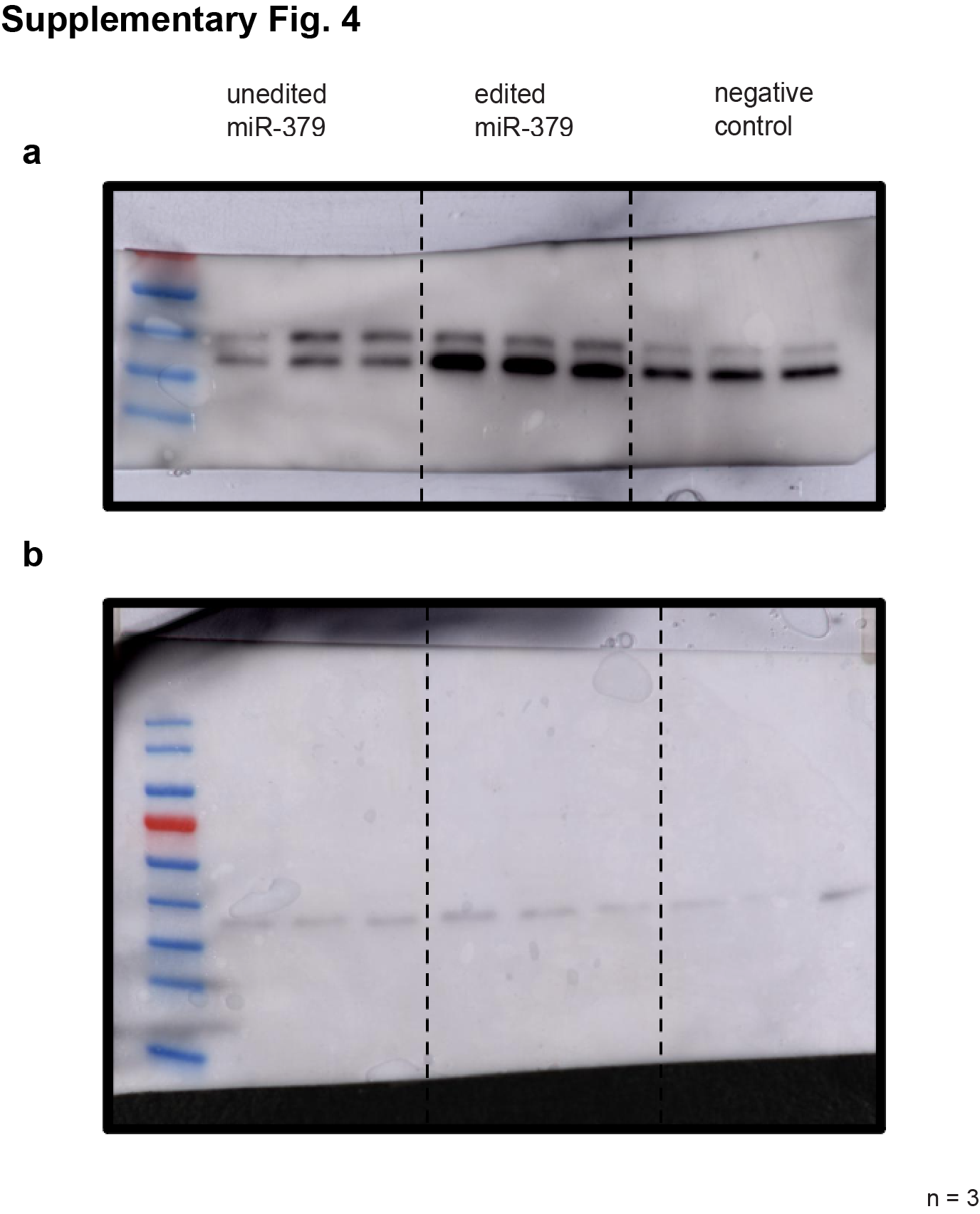


**Supplementary Figure 4.** Deglycosylation of protein lysates. PC3 lysates were deglycosylated prior to Western blotting of the untreated (**A**) and PNGase F-treated (**B**) lysates. The shown images are representative of three independent repeats.


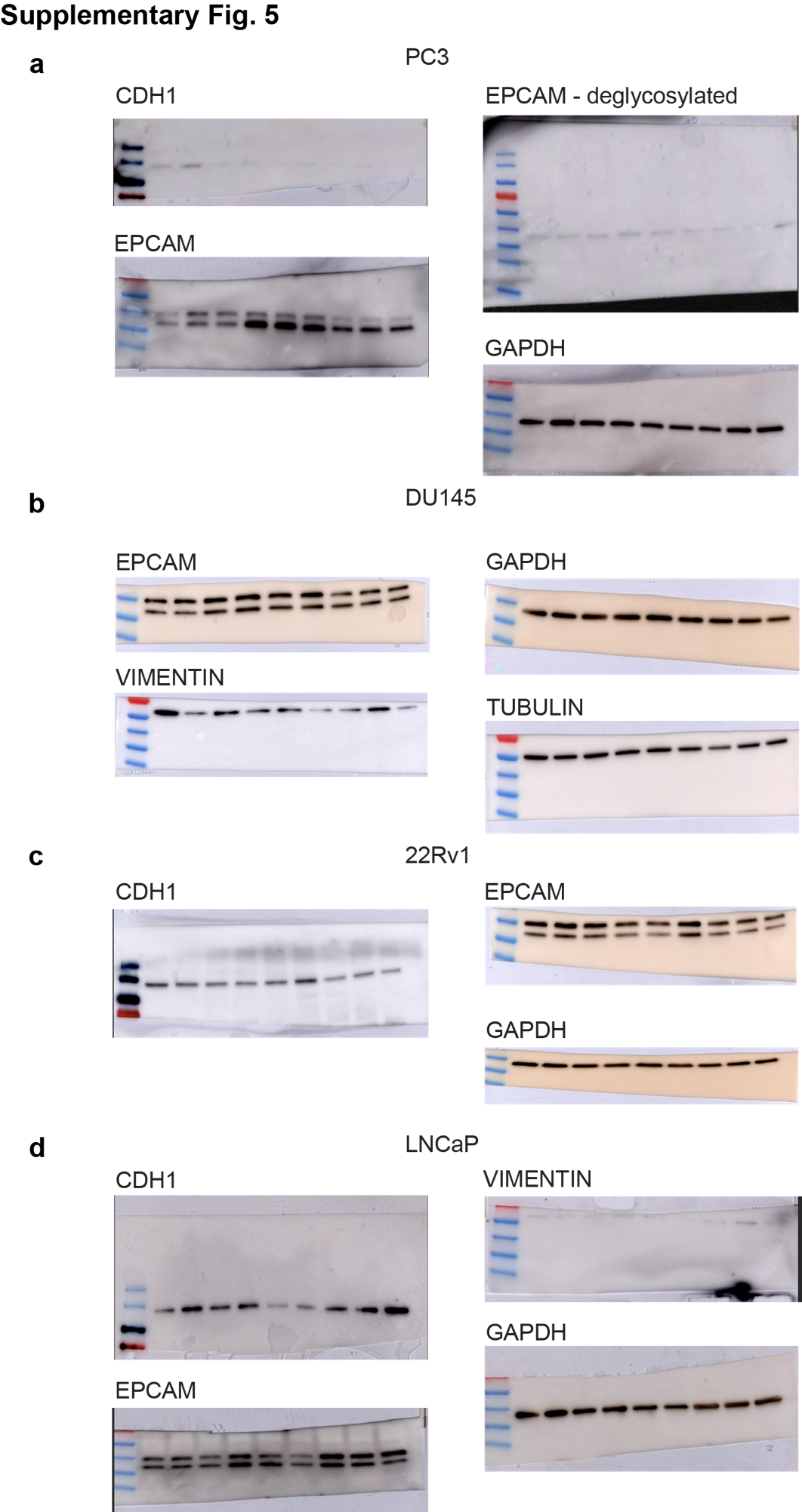


**Supplementary Figure 5.** Uncropped Western blots. The original JPEG files are attached from the selected representative Western blots for PC3 (**A**), DU145 (**B**), 22Rv1 (**C**) and LNCaP (**D**) cell lysates. Original membranes from gel were cut at the 70 kDa red band with CDH1 bands in the upper part of membrane being shown and EPCAM, Vimentin, GAPDH and Tubulin in the lower section of membrane. To save time and unnecessary stripping of the membranes which leads to increased protein degradation the membranes were cut before primary antibodies were added, with the exception of the deglycosylated EPCAM.


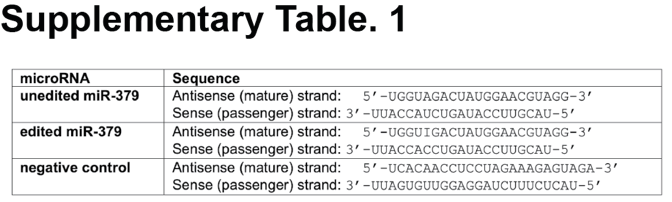


**Supplementary Table 1:** Sequences of microRNA mimics used in the study. Antisense strand and sense strand sequences of unedited miR-379, edited miR-379 and the negative control.


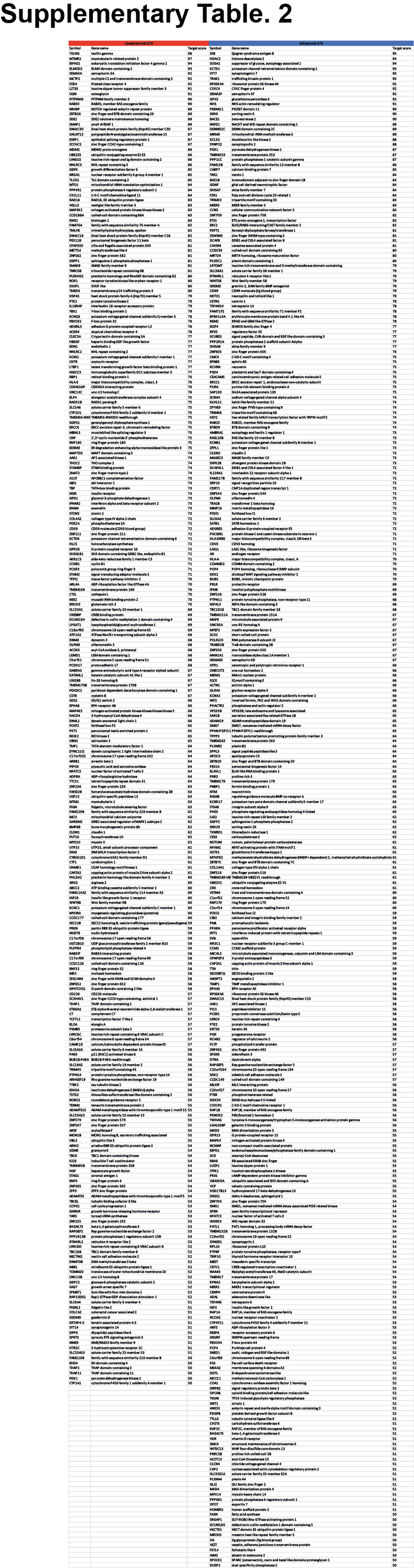


**Supplementary Table 2:** Table of all predicted targets for unedited and edited miR-379. The predicted targets are shown with descending target scores as predicted by miRDB.org.
